# Supplementary material for: Transmission, localization, and infectivity of seedborne maize chlorotic mottle virus
Source: PLoS One. 2023 Feb 6;18(2):e0281484. doi: 10.1371/journal.pone.0281484 (PMC9901749; doi:10.1371/journal.pone.0281484)
Supplement: S1 Table — (DOCX) [file pone.0281484.s001.docx]

**Supplemental Table S1. Sensitivity of ELISA for detection of Maize chlorotic mottle virus (MCMV).**

| **Leaf Punch Ratio MCMV:Healthy^a^** | **Replicates** | **MCMV detected** |
| --- | --- | --- |
|  |  |  |
| 1:11 | 5 | + |
| 1:15 | 2 | + |
| 1:24 | 2 | + |
| 1:23 | 5 | + |
| 1:25 | 2 | + |
| 1:30 | 2 | + |
| 1:35 | 2 | + |

^a^A single 0.25 in leaf punch from a maize plant (Oh28) infected with MCMV-KS was added to the indicated number of leaf punches were collected MCMV plants. Samples were extracted and used for DAS-ELISA as outlined in the Materials and Methods. Pools were considered positive (+) if the absorbance at 405 nm was greater than twice the mean absorbance of healthy controls.
