# Supplementary material for: Transmission, localization, and infectivity of seedborne maize chlorotic mottle virus
Source: PLoS One. 2023 Feb 6;18(2):e0281484. doi: 10.1371/journal.pone.0281484 (PMC9901749; doi:10.1371/journal.pone.0281484)
Supplement: S2 Table — (DOCX) [file pone.0281484.s002.docx]

**Supplemental Table S2. Efficiency of Maize chlorotic mottle virus (MCMV)-KS transmission by vascular puncture inoculation (VPI).**

| **Virion concentration^a^** | **Germination frequency** | **Transmission frequency** |
| --- | --- | --- |
| µg/µl | (%) | (%) |
| 1.00E-01 | 57 ± 32 | 100 ± 0 |
| 1.00E-02 | 80 ± 20 | 97 ± 6 |
| 1.00E-03 | 83 ± 12 | 96 ± 6 |
| 1.00E-04 | 90 ± 17 | 85 ± 15 |
| 1.00E-05 | 87 ± 15 | 35 ± 7 |
| 1.00E-06 | 83 ± 15 | 14 ± 15 |

^a^The transmissibility of MCMV was evaluated at six virion concentrations (determined using BioRad protein assay, Bernardo et al. 2021) to evaluate transmission frequency in three experiments. MCMV was diluted in phosphate buffer or the negative seed soak solution from seed prepared for VPI.
