# Supplementary material for: Transmission, localization, and infectivity of seedborne maize chlorotic mottle virus
Source: PLoS One. 2023 Feb 6;18(2):e0281484. doi: 10.1371/journal.pone.0281484 (PMC9901749; doi:10.1371/journal.pone.0281484)
Supplement: S3 Table — (DOCX) [file pone.0281484.s003.docx]

**Supplemental Table S3. Transmissibility of Maize chlorotic mottle virus (MCMV) from seed harvested from MCMV-HI infected plants at three maturity stages.**

| **Seed lot^a^** | **Moisture content^b^** | **Germination frequency** | **Transmission frequency^c^** |
| --- | --- | --- | --- |
|  | (%) | (%) | (%) |
| CML333 R2 | - | 65 | 84 |
| CML333 R4 | - | 66 | 43 |
| CML333 R6 | 23 ± 0.4 | 80 | 41 |
| CML333 R6 | 17 ± 0.3 | 84 | 5 |
| CML333 R6 | 14 ± 0.2 | 78 | 0 |
| CML545 R2 | - | 88 | 67 |
| CML545 R4 | - | 59 | 72 |
| CML545 R6 | 24 ± 0.4 | 65 | 0 |
| CML545 R6 | 17 ± 0.2 | 71 | 0 |
| CML545 R6 | 14 ± 0.1 | 73 | 0 |
| Positive control | - | 83 | 60 |
| Negative control | - | 80 | 0 |

^a^Cobs were collected from MCMV infected plants grown in Hawaii.

^b^Seed from R6 cobs was dried to the indicated moisture content as described in the supplemental methods.

^c^Transmission frequency of MCMV from seed extracts to MCMV-free seed by vascular puncture inoculation is shown. R6 seed was dried to the desired moisture content to assess its impact on MCMV transmissibility.
