# Supplementary material for: Transmission, localization, and infectivity of seedborne maize chlorotic mottle virus
Source: PLoS One. 2023 Feb 6;18(2):e0281484. doi: 10.1371/journal.pone.0281484 (PMC9901749; doi:10.1371/journal.pone.0281484)
Supplement: Attachment — Submitted filename: Response to reviewers Final.docx [file pone.0281484.s004.docx]

PONE-D-22-27020

Transmission, localization, and infectivity of seedborne Maize chlorotic mottle virus

PLOS ONE

Dear Dr. Erik Ohlson,

Thank you for submitting your manuscript to PLOS ONE. After careful consideration, we feel that it has merit but does not fully meet PLOS ONE’s publication criteria as it currently stands. Therefore, we invite you to submit a revised version of the manuscript by 31 December 2022, that addresses the points raised during the review process.

We look forward to receiving your revised manuscript.

Kind regards,

Islam Hamim, PhD
Academic Editor
PLOS ONE

Journal Requirements:

When submitting your revision, we need you to address these additional requirements.

1. Please ensure that your manuscript meets PLOS ONE's style requirements, including those for file naming. The PLOS ONE style templates can be found at 
<https://journals.plos.org/plosone/s/file?id=wjVg/PLOSOne_formatting_sample_main_body.pdf> and 
<https://journals.plos.org/plosone/s/file?id=ba62/PLOSOne_formatting_sample_title_authors_affiliations.pdf>.

Minor formatting changes have been made to meet PLOS ONE style requirements.

2. We note that the grant information you provided in the ‘Funding Information’ and ‘Financial Disclosure’ sections do not match.

The ‘Financial Disclosure’ section has been updated to match the ‘Funding Information’ section.

When you resubmit, please ensure that you provide the correct grant numbers for the awards you received for your study in the ‘Funding Information’ section.

3. We note that you have included the phrase “data not shown” in your manuscript. Unfortunately, this does not meet our data sharing requirements. PLOS does not permit references to inaccessible data. We require that authors provide all relevant data within the paper, Supporting Information files, or in an acceptable, public repository. Please add a citation to support this phrase or upload the data that corresponds with these findings to a stable repository (such as Figshare or Dryad) and provide and URLs, DOIs, or accession numbers that may be used to access these data. Or, if the data are not a core part of the research being presented in your study, we ask that you remove the phrase that refers to these data.

We apologize for the error. The data are reported in the text. Supporting tables/figures would not be informative since there is no fluorescence in the images. Data not shown has been removed to meet PLOS ONE guidelines.

4. Please include captions for your Supporting Information files at the end of your manuscript, and update any in-text citations to match accordingly. Please see our Supporting Information guidelines for more information: <http://journals.plos.org/plosone/s/supporting-information>.

Captions have been added to each of the Supporting Information Tables at the end of the manuscript.

5. Please review your reference list to ensure that it is complete and correct. If you have cited papers that have been retracted, please include the rationale for doing so in the manuscript text, or remove these references and replace them with relevant current references. Any changes to the reference list should be mentioned in the rebuttal letter that accompanies your revised manuscript. If you need to cite a retracted article, indicate the article’s retracted status in the References list and also include a citation and full reference for the retraction notice.

Minor stylistic changes have been made to the references to meet PLOS ONE style requirements.

Additional Editor Comments:

Revision is required based on suggestions from the reviewers.


[Note: HTML markup is below. Please do not edit.]

Reviewers' comments:

Reviewer's Responses to Questions

**Comments to the Author**

1. Is the manuscript technically sound, and do the data support the conclusions?

The manuscript must describe a technically sound piece of scientific research with data that supports the conclusions. Experiments must have been conducted rigorously, with appropriate controls, replication, and sample sizes. The conclusions must be drawn appropriately based on the data presented.

Reviewer #1: Partly

Reviewer #2: Yes

2. Has the statistical analysis been performed appropriately and rigorously?

Reviewer #1: Yes

Reviewer #2: No

3. Have the authors made all data underlying the findings in their manuscript fully available?

The [PLOS Data policy](https://gcc02.safelinks.protection.outlook.com/?url=http%3A%2F%2Fwww.plosone.org%2Fstatic%2Fpolicies.action%23sharing&data=05%7C01%7C%7Cda45ceadc2b24ba617f108dac7e910d3%7Ced5b36e701ee4ebc867ee03cfa0d4697%7C0%7C0%7C638042101431987028%7CUnknown%7CTWFpbGZsb3d8eyJWIjoiMC4wLjAwMDAiLCJQIjoiV2luMzIiLCJBTiI6Ik1haWwiLCJXVCI6Mn0%3D%7C3000%7C%7C%7C&sdata=GPaWWuC46w3ALK5NdVvXb77ELS9EvWC55cSKVusG5qM%3D&reserved=0) requires authors to make all data underlying the findings described in their manuscript fully available without restriction, with rare exception (please refer to the Data Availability Statement in the manuscript PDF file). The data should be provided as part of the manuscript or its supporting information, or deposited to a public repository. For example, in addition to summary statistics, the data points behind means, medians and variance measures should be available. If there are restrictions on publicly sharing data—e.g. participant privacy or use of data from a third party—those must be specified.

Reviewer #1: Yes

Reviewer #2: Yes

4. Is the manuscript presented in an intelligible fashion and written in standard English?

PLOS ONE does not copyedit accepted manuscripts, so the language in submitted articles must be clear, correct, and unambiguous. Any typographical or grammatical errors should be corrected at revision, so please note any specific errors here.

Reviewer #1: Yes

Reviewer #2: Yes

5. Review Comments to the Author

Please use the space provided to explain your answers to the questions above. You may also include additional comments for the author, including concerns about dual publication, research ethics, or publication ethics. (Please upload your review as an attachment if it exceeds 20,000 characters)

Reviewer #1: The manuscript is a detailed study aiming to clarify the factors underlying the transmission process of MCMV, which along with infection by a potyvirus is a threat for maize production worldwide. The scientific soundness of the paper is not new, as most of the results confirm previous studies on the subject, but it clarifies some factors involved in the infectivity of seed-born MCMV.
I trust an important aspect involved in the seed transmission might be the virus strain and this should be more carefully detailed and discussed in the manuscript. How are considered the two MCMV strains used in this work? Are they mild or severe strains? This could be a factor influencing the contrasting seed transmission rates found between this work and the one by Quito Avila et al. 2016.
Despites quite well written, I will try to contribute by suggesting minor modifications.

Thank you for your thorough review and thoughtful comments. Maize chlorotic mottle virus has minimal genetic diversity. While it is possible that there are minor differences in MCMV severity between the Hawaii, Kansas, and Kenya isolates used in this study we do not see evidence that virus strain would explain the seed transmission discrepancies between the Quito-Avila study and other studies that have found very low transmission rates. Comparisons of Hawaii and Ecuadorian MCMV isolates found that they clustered in the same phylogenetic clade and there was just 0.01 nucleotide diversity among this group (Braidwood et al. 2018). We are unaware of any major differences in severity among MCMV strains. Some additional exploration of these factors has been added to the discussion.

Line 25. Follow ICTV rules for virus name citation; first letter should not be capitalized here.

Thank you for bringing this to our attention. The manuscript has been revised to follow ICTV rules.

Line 44. Virus species name must be italicized.

Virus species is now italicized.

Lines 78-81. I trust this is Material and Methods.

Lines 107. Can light intensity be expressed in μmol?

The light intensity units are now given in the appropriate SI unit (candela)

Lines 108-109. Why different maize genotypes were used as positive and negative controls? They should carry the same genetic background to offer a uniform basis for evaluating symptoms.

Early Sunglow was selected based on the commercial availability of this variety and high MCMV susceptibility. Individuals in the growout tests were evaluated on a presence/absence basis by serological tests so variation in symptomology would not impact the results presented in this manuscript.

Line 241. “none was”.

“None were” is correct grammatically since it is referring the plural “pools”

Line 252. Delete repeated “in no washed samples”.

Thank you. The redundancy has been addressed.

Line 310. Suggestion: “for mock-inoculation with buffer”.

This is a good suggestion and we have incorporated it into the manuscript.

Line 318. Add “these” before “cells”.

Thank you, we agree with the recommended edit.

Line 400. Delete “in this lot”.

Thank you. We agree this is redundant and have deleted it.

Reviewer #2: This timely paper helps to clarify important gaps in understanding of seed transmission in MCMV with implications for other seedborne viruses. Quantifying seed transmission risk is complicated and fraught with pitfalls; I compliment the authors for the thoroughness of the work presented here and their thoughtful interpretations. I have only a few mostly minor comments, but one recommendation that is more important is related to the way the seed transmission frequencies are calculated, which I believe has led to overestimates.

Thank you for the thoughtful review and excellent comments. As you point out, the transmission frequencies were likely overestimates. Based on your recommendation, new estimates have been calculated using appropriate statistical analyses and 95% confidence intervals have been provided.

Line 61 – The wording of this sentence is awkward; “crop virus epidemics” could be revised to “virus epidemics in crops.”

The suggested edit has been incorporated.

Line 140 – More details should be described about exactly how the tissue dissections were carried out. Some of this appears in the results section, but I think it should be described here. As the authors acknowledge, there is some risk of cross-contamination during this procedure, so it is important for the reader to understand the details of how it was performed.

Additional details have been added to provide a more thorough description of the dissection.

Line 145 – Were embryos or other tissues pooled from multiple seeds before the ELISA testing? Or were individual embryos, etc., tested?

Each tissue sample was processed individually and tested by ELISA. The manuscript has been revised to more clearly indicate this.

Lines 172-189 – This section lacks a description of the VPI procedure or even a reference to how it is performed. These are important details; a brief description and reference should be added.

Thank you, we agree with the recommendation. We added a brief description in addition to the previously cited reference.

Line 202 – This sentence is not informative without first understanding what Figure 1 presents. I suggest revising it to say, “The relative MCMV titer among different seed tissues was determined…(Fig. 1).”

Thank you for the suggestion. The manuscript has been revised accordingly.

Table 1 – “Emergence” would be more accurate than “germination.” Germination is a specific seed quality measurement that is measured under standard conditions, following established guidelines. The data shown here are for emergence under the conditions of the experiment.

Thank you for the clarification. Germination has been changed to emergence throughout the manuscript.

Line 234 and Line 244 - Transmission frequency can be calculated and presented in a more accurate and precise way. It is possible to calculate a confidence interval around the estimates that are >0, and the ranges given for CML545 from Hawaii and the total of all seed lots are too high at the upper end. The upper bound of these estimates are based on the assumption that it would be possible that all seedlings in a positive pool might be positive (line 232). However, that possibility is exceedingly small, especially considering that 2,898 of the 2,900 pools (CML545) were negative. Assuming a binomial distribution of testing results (each pool can only be either positive or negative), with approximately 2,900 pools of 10 seeds each, if 2 pools are positive, the estimate of seed transmission frequency is indeed about 0.007% as reported by the authors. Tools are available to calculate confidence intervals around that estimate and by my calculations, the upper bound of the 95% CI for this estimate should be about 0.022%, NOT 0.07%. The probability that all twenty seedlings in both pools were infected (0.07% transmission frequency) is very low, about 0.00006%, or a 99.99994% CI. Similarly, the upper bound (95% CI) for the estimate of transmission frequency for CML442 from Kenya should be 0.031%, and for all 85,163 seeds, it should be 0.009%, not 0.02%.

Thank you for the recommendation. Results have been updated with new estimates of the transmission frequency using appropriate statistical analyses. These new transmission frequencies should more accurately represent the true transmission frequencies.

Line 250 – Is “washing” the most appropriate term for this procedure? I would call it “disinfection” or “disinfestation.”

We added the “disinfestation” term to the manuscript to provide additional clarity regarding the purpose of the washing procedure.

Line 285 – It would be preferable to include these results in Table 2 (even though they are all zeroes). That table will be used in the future to illustrate your results, and if there is no mention of embryo or endosperm tissues in the table, the take-home message could be lost.

Columns have been added to table 2 for embryo and endosperm.

Line 316 – Was VPI performed on mature seeds? A description of the procedure would clarify this.

Yes, the M&M now includes a more detailed description of the protocol.

Line 330 – These percentages are based on a total of how many seedlings?

The number of seedlings has been added for each treatment.

Line 363 – It’s not clear what you mean by “conservative.” If you mean the estimates may be higher than actuality, I agree. Anyway, “conservative” could be interpreted by some readers as “low,” which is not what I think you mean.

Wording has been revised to avoid confusion.

Line 377 – There seems to be a word missing from this sentence.

Additional words have been added to improve clarity.

6. PLOS authors have the option to publish the peer review history of their article ([what does this mean?](https://gcc02.safelinks.protection.outlook.com/?url=https%3A%2F%2Fjournals.plos.org%2Fplosone%2Fs%2Feditorial-and-peer-review-process%23loc-peer-review-history&data=05%7C01%7C%7Cda45ceadc2b24ba617f108dac7e910d3%7Ced5b36e701ee4ebc867ee03cfa0d4697%7C0%7C0%7C638042101431987028%7CUnknown%7CTWFpbGZsb3d8eyJWIjoiMC4wLjAwMDAiLCJQIjoiV2luMzIiLCJBTiI6Ik1haWwiLCJXVCI6Mn0%3D%7C3000%7C%7C%7C&sdata=ytgsrjvY2%2BiHa2OtT1xutK54dA%2FluaTqGsi0HKT4krs%3D&reserved=0)). If published, this will include your full peer review and any attached files.


If you choose “no”, your identity will remain anonymous but your review may still be made public.


**Do you want your identity to be public for this peer review?** For information about this choice, including consent withdrawal, please see our [Privacy Policy](https://gcc02.safelinks.protection.outlook.com/?url=https%3A%2F%2Fwww.plos.org%2Fprivacy-policy&data=05%7C01%7C%7Cda45ceadc2b24ba617f108dac7e910d3%7Ced5b36e701ee4ebc867ee03cfa0d4697%7C0%7C0%7C638042101431987028%7CUnknown%7CTWFpbGZsb3d8eyJWIjoiMC4wLjAwMDAiLCJQIjoiV2luMzIiLCJBTiI6Ik1haWwiLCJXVCI6Mn0%3D%7C3000%7C%7C%7C&sdata=kYvt%2Fe8jNBcNuz4lwZ05AFlKhXnDzfXs5uSIMbKWEeg%3D&reserved=0).

Reviewer #1: No

Reviewer #2: **Yes:** Gary Munkvold

[NOTE: If reviewer comments were submitted as an attachment file, they will be attached to this email and accessible via the submission site. Please log into your account, locate the manuscript record, and check for the action link "View Attachments". If this link does not appear, there are no attachment files.]

While revising your submission, please upload your figure files to the Preflight Analysis and Conversion Engine (PACE) digital diagnostic tool, [https://pacev2.apexcovantage.com/](https://gcc02.safelinks.protection.outlook.com/?url=https%3A%2F%2Fpacev2.apexcovantage.com%2F&data=05%7C01%7C%7Cda45ceadc2b24ba617f108dac7e910d3%7Ced5b36e701ee4ebc867ee03cfa0d4697%7C0%7C0%7C638042101431987028%7CUnknown%7CTWFpbGZsb3d8eyJWIjoiMC4wLjAwMDAiLCJQIjoiV2luMzIiLCJBTiI6Ik1haWwiLCJXVCI6Mn0%3D%7C3000%7C%7C%7C&sdata=818E7So1XPG5Rb5ozDlWMlWEe1L7smdHMAwOtzjXu1M%3D&reserved=0). PACE helps ensure that figures meet PLOS requirements. To use PACE, you must first register as a user. Registration is free. Then, login and navigate to the UPLOAD tab, where you will find detailed instructions on how to use the tool. If you encounter any issues or have any questions when using PACE, please email PLOS at [figures@plos.org](mailto:figures@plos.org). Please note that Supporting Information files do not need this step.

*In compliance with data protection regulations, you may request that we remove your personal registration details at any time.* [*(Remove my information/details)*](https://gcc02.safelinks.protection.outlook.com/?url=https%3A%2F%2Fwww.editorialmanager.com%2Fpone%2Flogin.asp%3Fa%3Dr&data=05%7C01%7C%7Cda45ceadc2b24ba617f108dac7e910d3%7Ced5b36e701ee4ebc867ee03cfa0d4697%7C0%7C0%7C638042101431987028%7CUnknown%7CTWFpbGZsb3d8eyJWIjoiMC4wLjAwMDAiLCJQIjoiV2luMzIiLCJBTiI6Ik1haWwiLCJXVCI6Mn0%3D%7C3000%7C%7C%7C&sdata=f18C57Eu5CGOI9JkUUPK9oDdKeviILlcK10y827HgeY%3D&reserved=0)*. Please contact the publication office if you have any questions.*
